# Dynamic analysis of NGO emergency relief goods supply: 2020 Hubei COVID-19 as a case

**DOI:** 10.3389/fpubh.2023.1037846

**Published:** 2023-02-07

**Authors:** Yi Lu, Yuhang Wang

**Affiliations:** ^1^Institute of Emergency Management, Sichuan University, Chengdu, China; ^2^Business School, Sichuan University, Chengdu, China

**Keywords:** COVID-19, relief goods supply, NGO (non-governmental organization), system dynamic (SD) model, emergency relief

## Abstract

**Intention:**

Global emergencies cause significant damage to lives, assets, and the economy. Therefore, the supply of relief goods is essential in emergency relief contexts, which is generally the function of non-government organizations (NGOs) as they have unique relief goods supply advantages. However, few studies have explored the influencing factors on NGO relief goods supply efficiency. To systematically explore the factors affecting supply efficiency, we aim to develop a supply chain model for simulating and providing policy suggestions.

**Method:**

Taking the 2020 Hubei COVID-19 as case study, this research developed a system dynamic (SD) model for the NGO relief supply system to evaluate and quantify the impact of factor changes on relief supplies.

**Conclusion:**

It was found that transportation and information delays aggravated the NGO emergency supply chain bullwhip effect and caused large supply fluctuations. The initial relief goods inventory was found to be a decisive factor in reducing shortages in disaster areas; however, government support was found to play only a limited role in reducing information and transportation delays.

**Value:**

This study enriches NGO emergency supply chain literature and provides suggestions for guiding NGO relief goods supplies in the future.

## 1. Introduction

The frequency and scale of humanitarian emergencies from natural disasters, war, and disastrous emergencies such as COVID-19 or terrorism have increased in recent years, with research suggesting that there may be more of such emergencies in the near future (1, 2). These emergencies have highlighted the significant gap in relief goods. For example, relief goods were in short supply during the 2004 Indian Ocean tsunami disaster (3), the 2010 Haiti earthquake (4), and the 2015 Nepalese earthquake (5). During COVID-19, the demand for medical and daily living supplies has also increased significantly (6). Immediately after the occurrence of an emergency in the response phase, it is vital that relief goods are delivered as quickly as possible to the victims in the affected regions (7) as shortages or delivery delays can result in serious losses (8, 9). Therefore, to save the lives of the people in the affected regions (10), relief goods supply is a vital part of emergency relief operations.

Providing relief goods and distributing them to affected areas are important emergency relief operation functions. While there are many relief goods providers, the primary contributors have generally been national government organizations (GO), such as the Federal Emergency Management Agency (FEMA) in the USA, NGOs (the Red Cross, community rescue teams, etc.), and private donors. These three entities have different purposes when providing relief goods to the affected areas and use different distribution channels (11). Of these providers, NGOs have been playing vital roles (12). For example, after the 2005 Hurricane Katrina, the 2008 Wenchuan earthquake, and the 2013 Haiyan typhoon, many NGOs provided emergency relief goods to the affected areas as part of the post-disaster relief and reconstruction (13–15). Compared with the GOs, NGOs are often more flexible in their disaster responses (16–18). For example, when a magnitude 9.0 earthquake struck East Japan on March 11, 2011, the Japanese Council of Social Welfare was supposed to immediately open and manage a disaster volunteer center; however, this was delayed by 1 month. Conversely, the NGOs took between 1 day and 1 week to establish emergency relief centers that provided food and trained rescue teams (19). Another example is the Bhuj earthquake in Turkey when the public rescue team could not access the distant village. However, NGOs managed to successfully reach the community and provide food and medical services (20). In 2004, one of the deadliest tsunamis hit the Indian Ocean and resulted in 227,898 missing persons (21). The Red Cross, Crescent Societies, and hundreds of NGOs responded rapidly using communication technology and modern transportation to rescue people and transport relief goods, which was in contrast to the slow bureaucratic and inefficient decision-making processes of the government (21).

Because of the increased frequency of emergency events, there has been a renewed research focus on emergency supply chains (22). After an emergency, relief goods need to be efficiently transported to the disaster area to reduce casualties. Najafi et al. (23) developed a dynamic earthquake dispatch and vehicle routing to assist disaster managers to plan their logistical activities in the earthquake response phase. Cao et al. (24) designed relief strategies based on a sustainability beneficiary perspective, for which a multi-objective mixed-integer nonlinear programming model was formulated, which was solved using a proposed genetic algorithm. Singh et al. (25) identified and analyzed relief supply chain resilience factors to ensure post-disaster supply capacities. Most previous relief goods supply research has tended to focus on emergency logistics, routing planning, and relief goods allocation. However, systematic research on the influences of the various supply effect factors is equally important.

NGOs and humanitarian supply chains build a “fairness” concept into their supply models (26). Therefore, most humanitarian supply chain research has focused on the last-mile distribution problem. For example, Rabta et al. (27) examined the use of drones for last-mile humanitarian logistics distribution, for which an optimization model was developed for the drone delivery of multiple lightweight relief item packages to remote locations within the disaster-prone area. Chen and Zhao (12) examined NGO capacity limitations and the relationships between GOs and NGOs, for which they modeled the relationships between received and donated relief goods quantities and developed a conditional earmarking donation strategy. Tatham and Blecken (28) empirical survey found that most NGOs did not adequately assess their supply chain performances and the associated logistics activities because they did not have the necessary capabilities. Therefore, to systematically resolve these practical NGO relief goods supply problems, as a case example, this paper employed system dynamics (SD) to study the NGO relief goods supply chains during the 2020 Hubei COVID-19 crisis.

SD is a pseudo-continuous modeling and simulation approach that has been widely used to holistically analyze complex, interdependent, and non-linear systems (29). SD can solve simultaneity (mutual causation) problems by updating all variables in small time increments, providing positive and negative feedback and time delays, and structuring the interactions and control. As the strength of the SD modeling approach is its ability to forecast system behavior beyond the problem symptoms, it has previously been employed to model supply chain systems and evaluate the policy effects on systems (30). Olivares-Aguila and ElMaraghy (31) used an SD framework to observe supply chain behavior and evaluate the disruption impacts, and Xu et al. (32) developed an SD model to assist in the post-seismic relief supply allocations in the Longmen Shan fault area where many destructive earthquakes have occurred. Because SD methods are able to simulate and analyze specific human and environmental characteristics and their relationships during emergency events, it was deemed suitable for an analysis of the complex relief goods supply chain system (33).

In July 2020, the research team was invited to review the joint NGO relief goods supply activities in Hubei, which provided us with a rare opportunity to examine the NGO emergency supply chain system and obtain primary data for the SD model. This study was focused on three key problems: (1) what impact do emergency uncertainties have on NGO relief goods supplies; (2) how do the various factors affect the NGO emergency supply chain; and (3) how can the impact of these adverse factors be mitigated.

Using SD Simulation, this study explored the key elements of the NGO relief goods supply system and the influence of different behaviors on system performance. The preliminary investigation highlighted many of the practical NGO relief goods supply problems; therefore, it is hoped that the developed model and our conclusions can assist in resolving these problems.

## 2. NGO relief goods supply in Hubei during the COVID-19 crisis

The new coronavirus (COVID-19) that appeared in Hubei, China, at the end of 2019 had infected more than 10,000 Chinese people by February 1, 2020 (34). To mitigate the COVID-19 outbreak, a closure strategy was implemented on January 23, 2020, in Hubei, with other parts of China imposing travel restrictions (35). These policies effectively reduced the increase in infected people but also resulted in severe goods supply problems in Hubei (36). The government allocated more than 66 billion CNY to contain the outbreak and delivered 20 tons of medical supplies per hour across the country to support the relief effort. At the same time, the NGOs were fundraising for an epidemic prevention and control (EPC) program (12), for which the government established a coordination center. Specifically, the charitable foundations collected and donated funding to support the NGOs, and some emergency management NGOs and public health NGOs assisted state agencies, medical institutions, and foundations by delivering materials and services and assisting in the EPC program. Community-based NGOs assisted the local government responses (37), and community volunteers provided material assistance and emotional social support (38), which specifically included materials collection, distribution, transportation, and community services such as purchasing, sterilization, and psychological counseling (39).

While these actions and relief goods greatly assisted the EPC program, there were many problems in the actual goods supply process. For example, the purchase of materials over the internet meant that some elderly groups were unable to source enough materials (40). Because of the insufficient emergency materials reserves, hospitals across the country, and especially those in Hubei where the situation was the most severe, were short of needed medical supplies, especially personal protective supplies such as medical protective clothing and N95 masks (41).

In July 2020, the research team went to Hubei to investigate the NGO relief goods supply process during COVID-19. Supported by the Hubei United Disaster Relief Welfare Network (HUDRWN), 50 HUDRWN NGO members were selected for interviews and questionnaires using stratified sampling, and descriptive statistics, reliability, validity, correlation, and difference analyses were conducted to determine the results. The research team examined the NGO EPC program activities in Hubei and found the following emergency goods supply problems. (1) Because of the traffic controls, there were only a limited number of vehicles authorized to transport goods, which meant that many goods were not delivered on time. (2) There was serious information asymmetry. When the goods collected by the NGOs were delivered, the receiving party's demand was saturated. (3) Because there were many different models and medical materials, the distribution was often incorrect.

These problems were not confined to the NGO relief goods transportation as they also affected the efficiency of the community EPC activities. Therefore, this study sought to systematically analyze the complete NGO emergency goods supply process to determine the key factors affecting its efficiency.

## 3. Methods

This section reviews the systems dynamics approach, outlines the data analysis procedures, and details the model development.

### 3.1. SD model development

**Figure 1:**
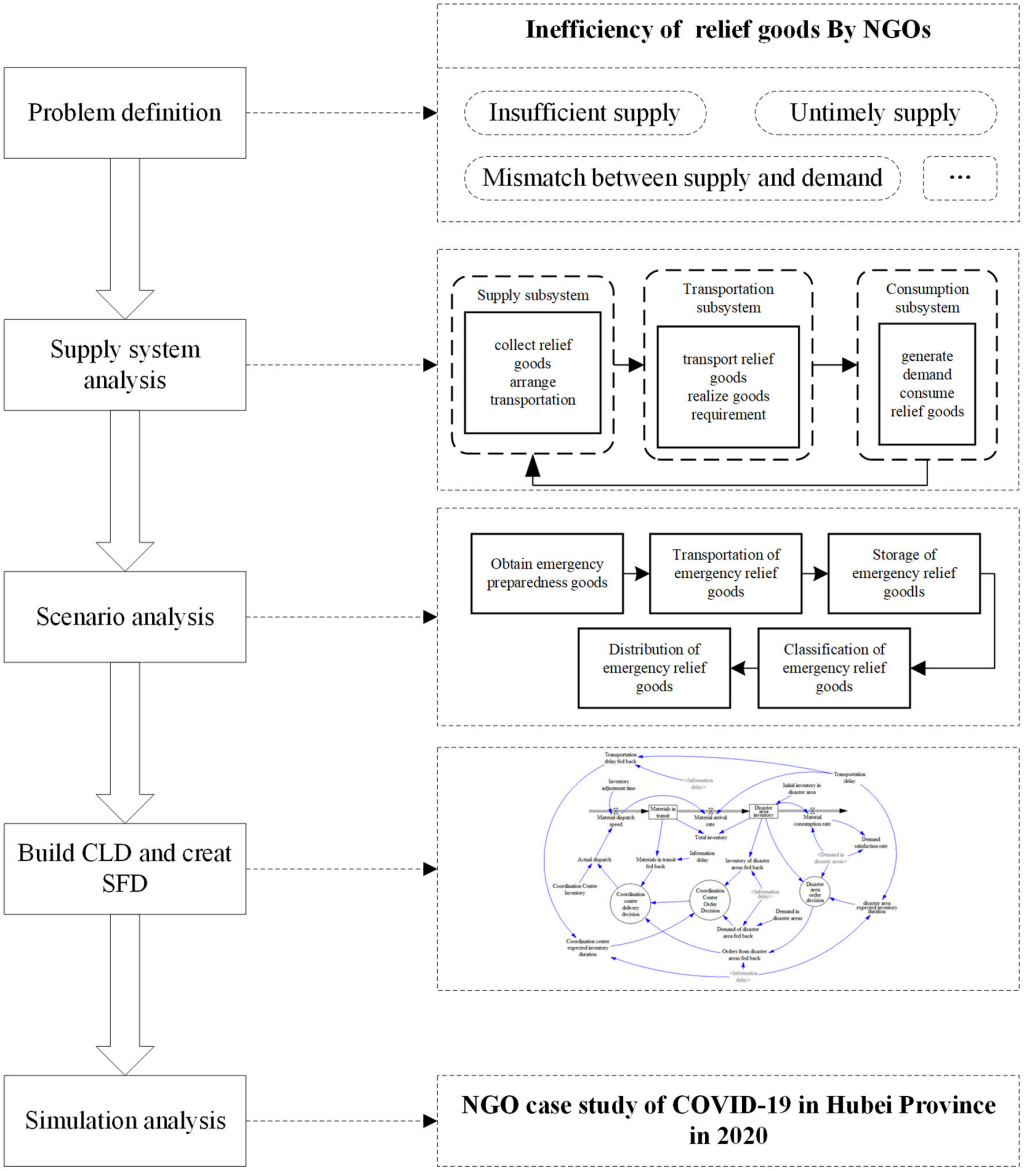
Model development algorithm.

The empirical SD research involved five consecutive steps (Figure 1): (1) problem definition; (2) systems analysis; (3) scenario analysis; (4) the building of the causal loop diagram (CLD) and the stock and flow diagram (SFD); and (5) conducting the simulation system analysis.

#### 3.1.1. System analysis

Environmental constraints regulate system behavior and system construction. Before describing the system structure, the supply system context needs to be further explored. As the NGO emergency relief goods dispatch and transportation is a complex process that had multiple links; materials procurement, collection, transportation, and distribution; it is necessary to balance the supply and the demand under resource, time, information, and transportation constraints. Therefore, it was necessary to first analyze the system structure and functions.

The NGO relief goods supply chain is a complete process from the receipt of goods to the final distribution to the victims, that is, the process involves relief goods acquisition, procurement, transportation, storage, sorting, and distribution.

**Figure 2:**
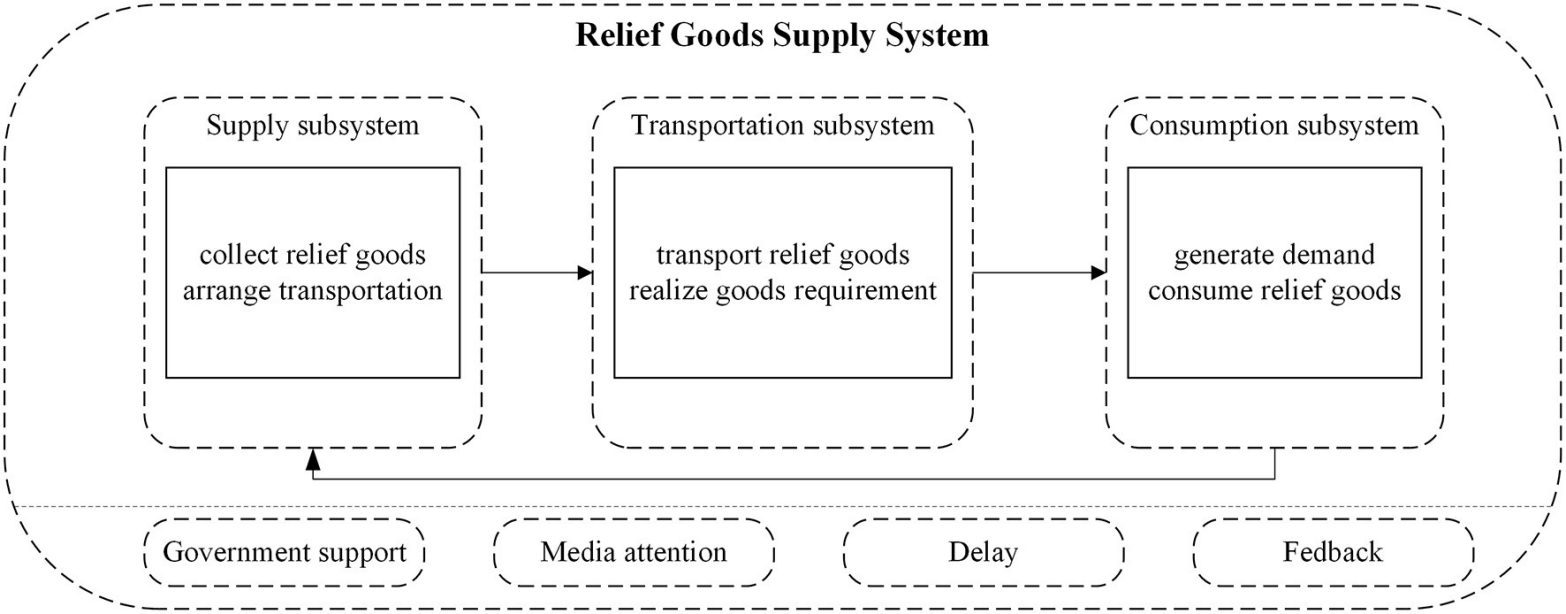
Supply system framework.

The system structure analysis examined all system elements, the interdependencies between these elements, and the mutual interactions. As dynamic behavior comprises information feedback, circular causalities, and the influences of these loops on the system variables (42), the supply system was divided into three subsystems: a supply subsystem; a transportation subsystem; and a consumption subsystem. The main task of the supply subsystem was to collect the relief goods and supply them as required, the actions of which were inevitably influenced by the government and media attention. The main transportation subsystem task was to transport the relief goods and feedback the actual disaster area needs to the NGOs; therefore, transportation and information delays needed to be accounted for. The main consumption subsystem function was to reflect the disaster area needs and the degree to which these needs were being met, as shown in Figure 2.

#### 3.1.2. Building the causal loop diagram

The dynamics of a system are related to the interactions between the reinforcing feedback loops and the balancing feedback loops (43). The feedback loop visualization embedded in the SD model is known as a causal loop diagram (CLD), the aim of which is to identify the interactions within the model components (44). The definitions for the components used for the CLD creation in this study to explain the dynamics that stimulated changes in the selected supply effect indicators are given in Table 1.

**Table 1:** CLD components.

**Component type**	**Description of purpose**	**Visual representation**
Variables	Indicates the variables in the system dynamic model	Text
Link	Indicates the links between the variables	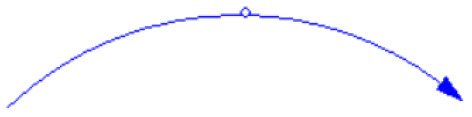
Link with time delay	Indicates the links between variables with a time delay	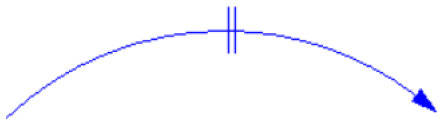
Reinforcing loop	Indicates that a loop has a reinforcing effect on the initial variable value	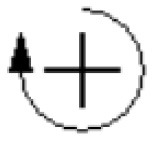
Balancing loop	Indicates that a loop has a balancing effect on the initial variable value	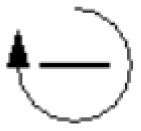

**Figure 3:**
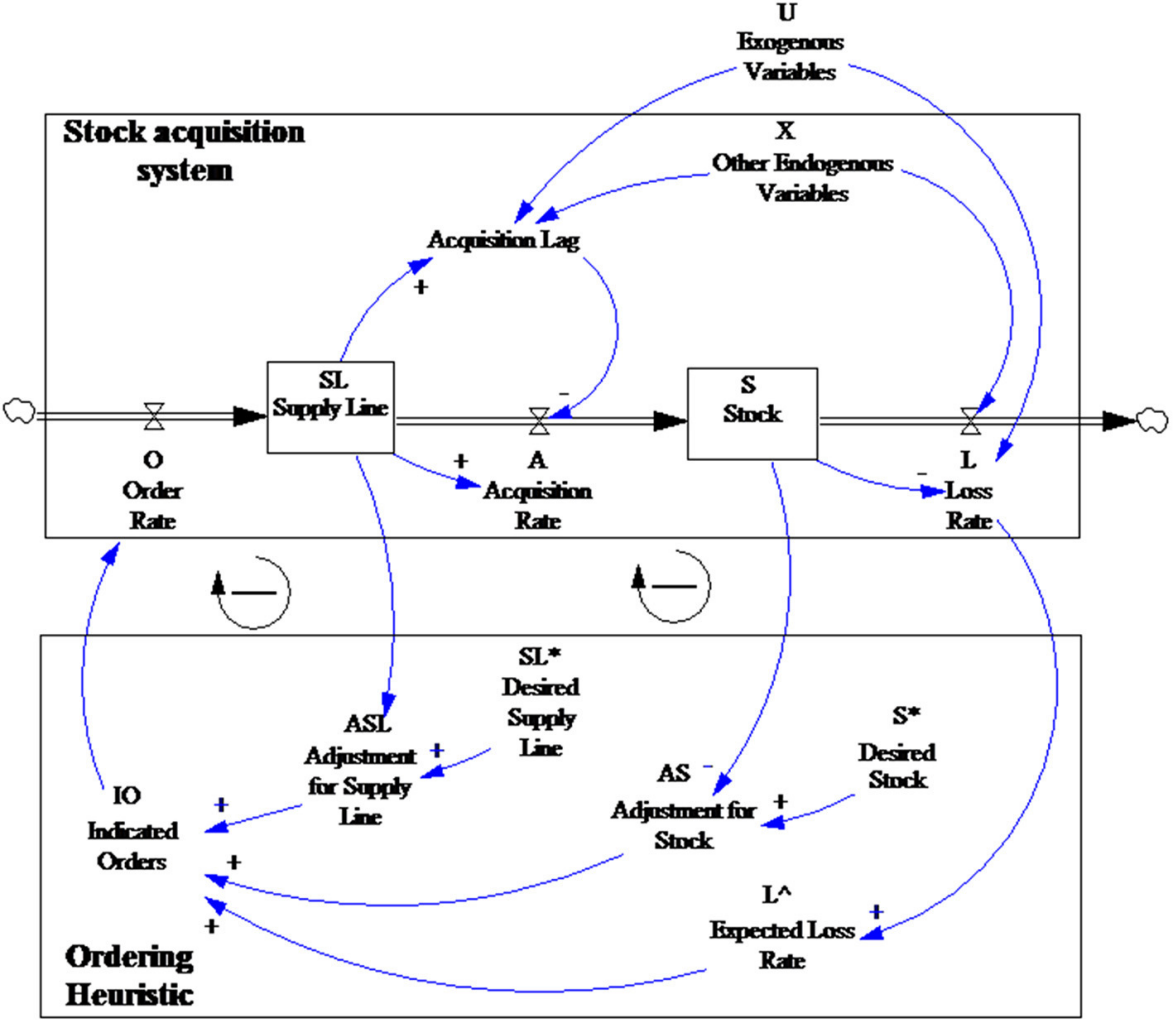
The generic stock-management system by Sterman.

Sterman (45) divided the generic stock management control problem into two parts: (1) a stock and flow system structure, and (2) a decision rule to be used by managers (Figure 3). There was one reinforcing loop and two balancing loops in the system. An increase in loss leads to a continuous increase in order and stock, which further increases the loss, which is the reinforcing loop. An increase in stock reduces the gap between stock and expected stock, reduces the adjustment for stock, and finally leads to a reduction in stock, which is the balancing loop. Similarly, the supply line reduces the gap between supply line and expected stock, which leads to a reduction in the adjustment for supply line, further reduces the order and finally leads to a reduction in the supply line, which is the balancing loop.

**Figure 4:**
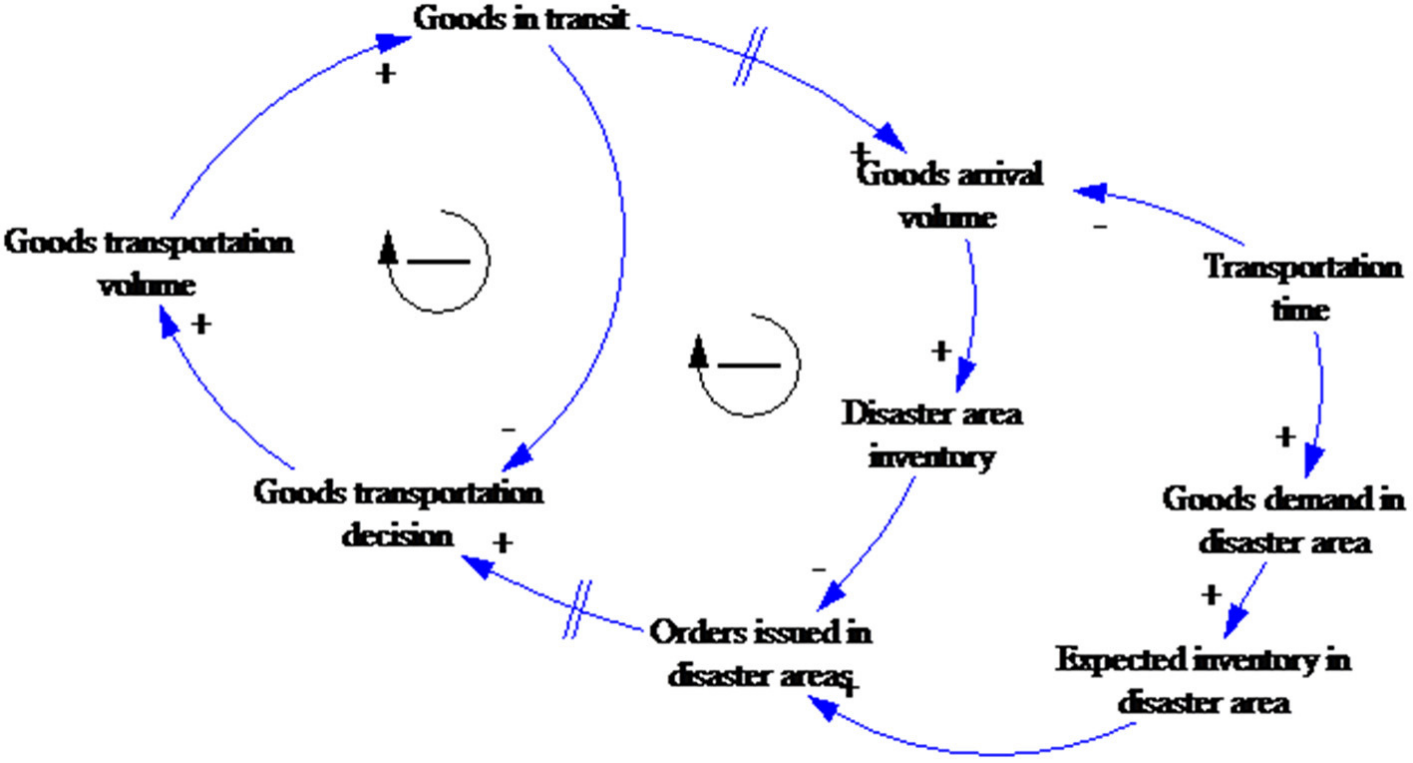
Relief goods supply system casual loop diagram.

As outlined in NGO relief goods supply in Hubei during the COVID-19 crisis section, compared with general storage and supply models, the NGO emergency goods supply is more specialized. First, the coordination center rather than a supplier makes the order decisions, which can result in information delays between the disaster areas, the coordination center, and NGOs. Second, the demand satisfaction rate for the relief goods supply is far more important than the other evaluation indicators. Third, the factors affecting supply and transportation are more complex, such as focused media attention and government support. This paper, therefore, took Sterman's generic stock-management system model as the framework and then combined it with the emergency relief goods supply characteristics to develop the causal circuit diagram shown in Figure 4.

The CLD shown in Figure 4 has two balancing loops representing the influence of the delivered goods on the goods transportation decision-making. The two links between goods transit and decision-making are marked for time delays depending on the delay characteristics, that is, material delays, information delays, or transit delays, such as volume restrictions, road conditions, government support, and other factors.

**Figure 5:**
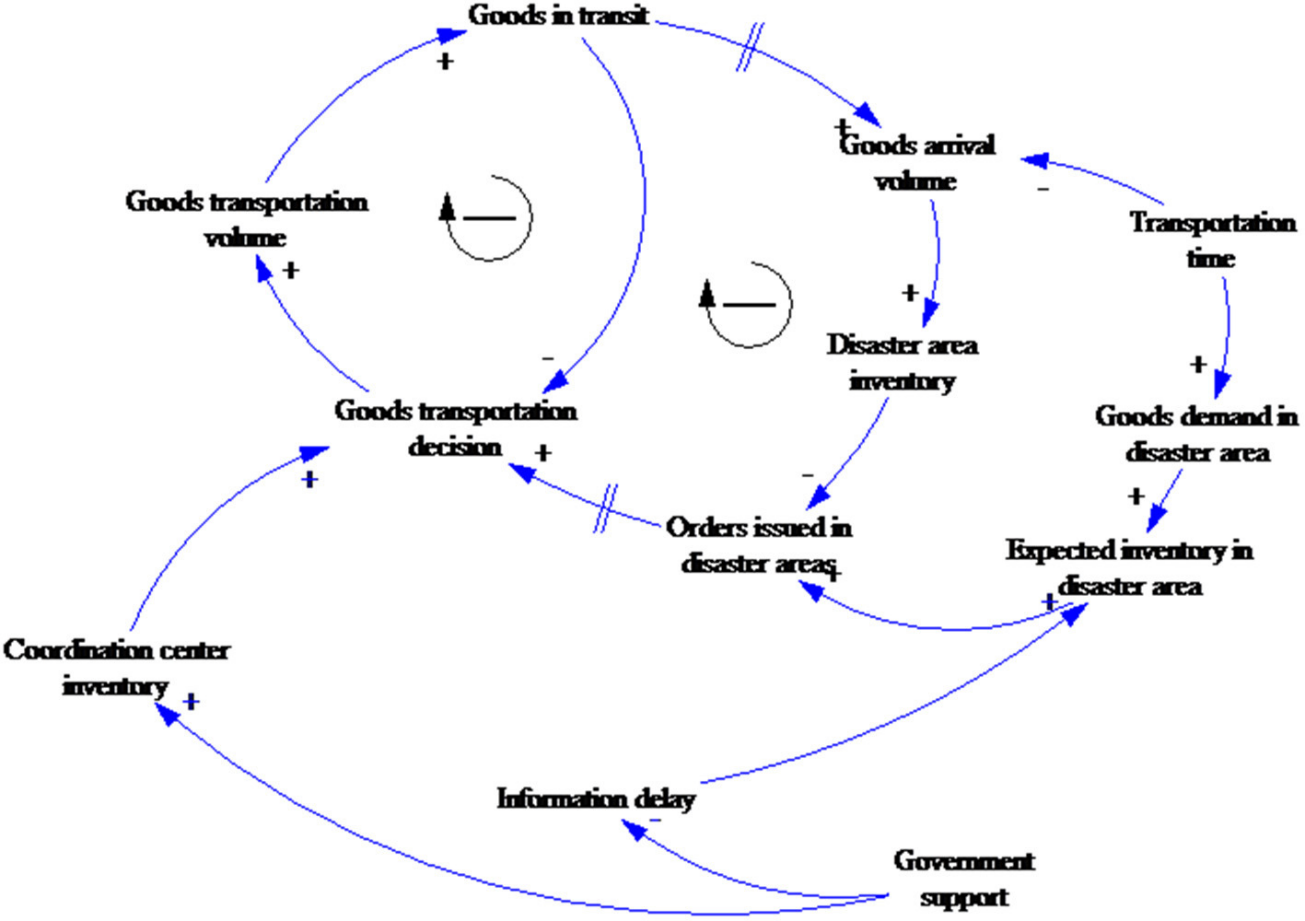
Relief goods supply system casual loop diagram (including government support).

As NGOs have accumulated emergency relief experience, NGOs in China and other countries have gradually formed NGO networks, which has significantly enhanced the ability of NGOs to obtain emergency relief information (46). However, because of limited resources and capacity, and the government's leading role as a most responsible emergency relief provider (47), if the level of government support is improved and a transparent, efficient information platform is established for the disaster areas and the NGOs, the information delay is significantly reduced. The CLD with government support is shown in Figure 5.

#### 3.1.3. Creating the stock and flow diagram

**Figure 6:**
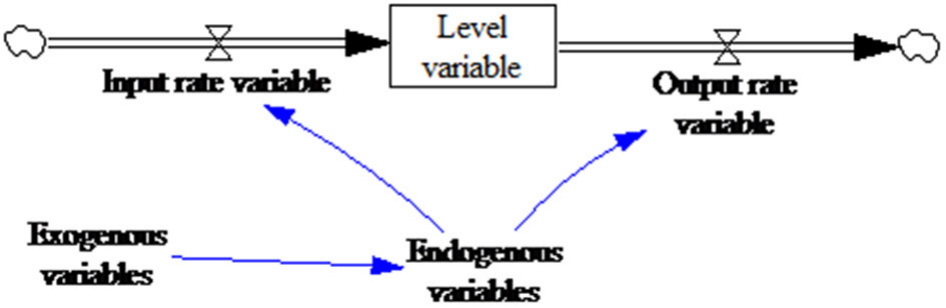
Stock and flow diagram symbols.

The SFD distinguishes the nature of each variable and describes the system elements and overall framework. Intuitive symbols depict the logical relationships between the elements to allow for an exploration of the system's feedback and control rules (48). In the system flow diagram shown in Figure 6, the level variables represented by the boxes reflect the system state, the values for which are determined from the materials accumulation and information flow results from an initial point to a particular moment, such as the goods in transit and the disaster area inventories. The rate variables reflect the system state changes per unit time, such as transportation delays, and the constant variables are the system's parameters, which change little over time scope and reflect certain elements, such as the consumption rate.

**Figure 7:**
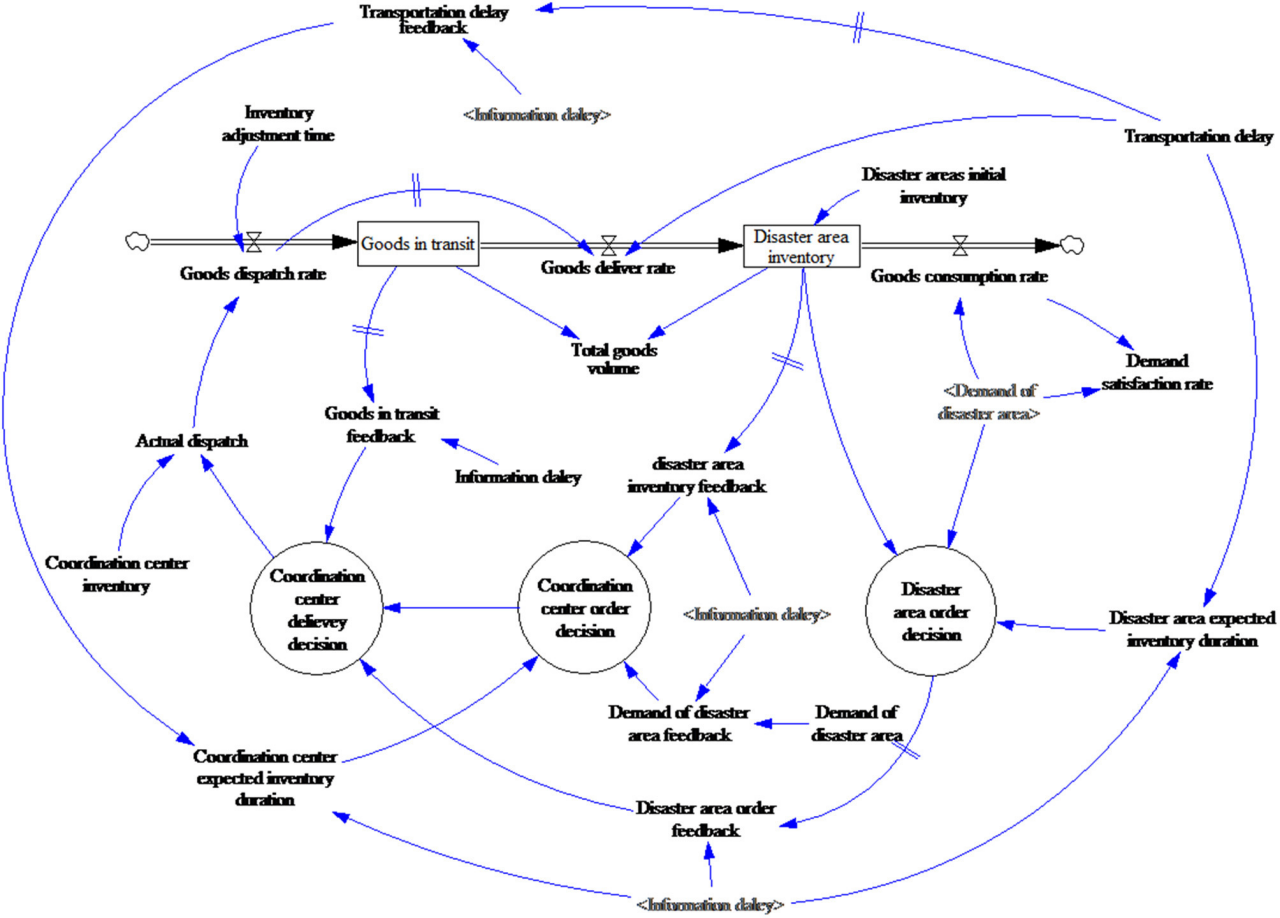
Relief goods supply system stock and flow diagram.

Based on the CLD, the resulting SFD is shown in Figure 7.

The relief goods volume is the core variable in the model. Depending on the relief scenario, the goods are divided into *goods in transit* and *disaster area inventory*. Goods dispatch and goods delivered, respectively result in increases and decreases in the *goods in transit*, and goods dispatch and goods consumption, respectively result in increases and decreases in the *disaster area inventory*.

The demand satisfaction rate is the core evaluation index for this model. Nutrition research has found that when the daily adult energy intake is <70% of the general level for some time, it has an adverse impact on health and can result in malnutrition and various diseases (49). Therefore, the time when satisfaction falls below 70% needs to be paid attention to.

The decision variable is the adjustable quantities in the actual decisions, which are represented by a circle in this model, such as the *coordination center delivery decision* and the *disaster area order decision*. The *coordination center delivery* volume is determined after the information delay by the *disaster area order decision* and the *coordination center order decision*.

There are two main NGO sources for relief goods and funds; donations from caring enterprises and people, and government service purchases. For the first source, the direct influencing factor is social donation enthusiasm, which is mainly related to the disaster severity, the number of affected people, and the media attention but can also be affected by government policies (support or restrictions). For instance, the release of Indian government documents improved the fund-raising capacities of local NGOs (50), and in another case study, NGO activities were found to be significantly restricted by a lack of *government support* (51). The second source of resources and funds is affected by the government's actions.

**Figure 8:**
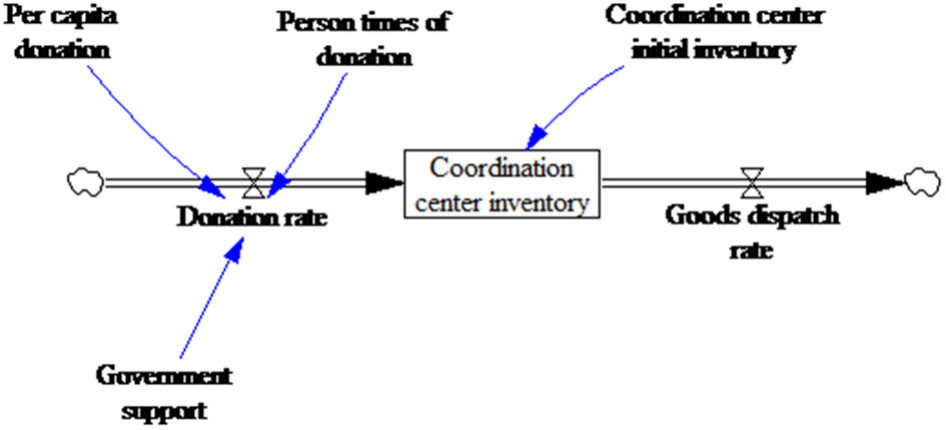
Donation dimension stock and flow diagram.

Therefore, because of donor and government influences, NGOs can experience goods supply discontinuities and uncertainties. Taking into consideration the above characteristics, a donation dimension SFD was built (Figure 8).

### 3.2. Data collection and analysis

Precise time series data are required to develop effective relief goods supply system dynamic models and test the accuracy of the model parameters. The required data were: (1) donation numbers and quantities in the period after the COVID-19 outbreak in Hubei; (2) the number of people affected by the pandemic and the goods consumption rate; and (3) the estimates for the road transport capacity and transportation and information delays.

**Figure 9:**
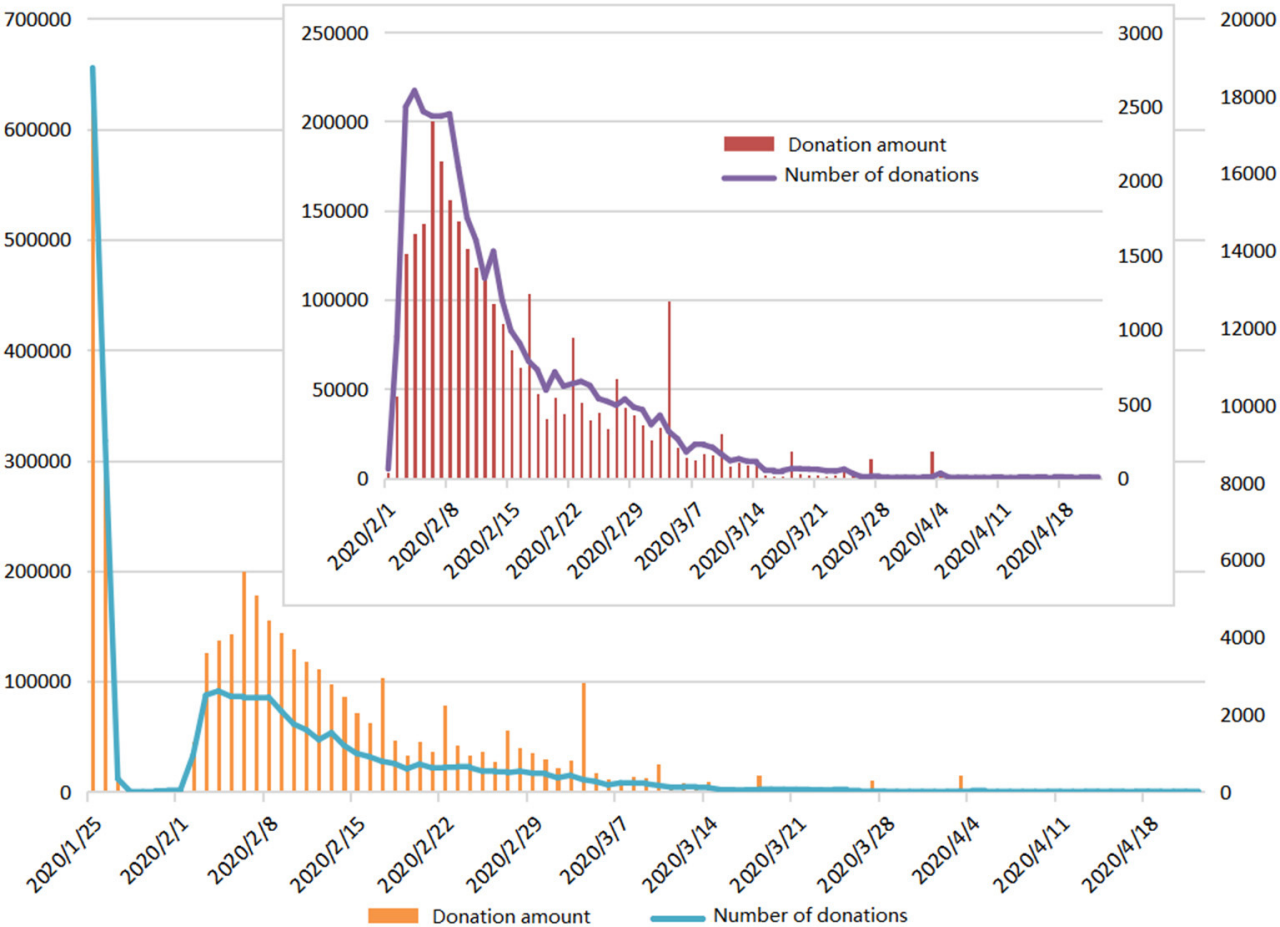
Hubei COVID-19 donation information.

To find the data for (1), a crawler was applied to the Baidu public donation information disclosure website (https://gongyi.baidu.com/dist/open-info.html) to obtain the donation information related to “COVID-19”, the results from which are shown in Figure 9.

Because of the Hubei lockdown that commenced on January 23 and attracted significant nationwide attention, there was a large number of donations from January 25 to 26. From January 25 to January 27, Baidu launched an “Aid Hubei EPC” fund-raising project, which received a large number of donations from caring people. When the interference of this factor was excluded, after February 1, the donations and the number of people related to COVID-19 peaked within a week and then began to slowly decline. With the input of rescue forces after the pandemic outbreak, the situation was gradually controlled, the demand in the pandemic area reduced, and public attention on COVID-19 gradually weakened, as shown in Figure 9.

Different from a general supply chain, the emergency relief supply chain has urgency and uncertainty characteristics. The relief goods can be divided into two categories: (i) cumulative demand, such as tents and disaster relief equipment, which if not met in time, increases the subsequent demand; and (ii) non-cumulative demand, such as drinking water and food, which is fixed in each time period, that is, the unmet demand does not accumulate.

To find the data for (2), previous studies have examined emergency relief goods demand based on disaster information and historical data (52–54). Because this study was focused on the impact of the dynamic disaster area environment on NGO relief goods supplies, only the non-cumulative demand was considered. Therefore, based on the research, a negative exponential function was used to describe the gradual decline in demand after the emergency, to which a random variable was added to represent the noise, as shown in (1).


(1) $$\begin{array}{l} \;EXP\left(-RAMP\left(\left\{slope\right\},\left\{start\right\},\left\{finish\right\}\right)\right)\;\\ {}*RANDOM\;NORMAL\left(\left\{\min\right\},\left\{\max\right\},\left\{mean\right\},\left\{stdev\right\},\left\{seed\right\}\right) \end{array}$$

When examining the data for (3), because NGO rescues are confronted with road and communication obstacles, the transportation and information delays are generally high. However, as actions are taken to repair the roads, these delays gradually decrease. Therefore, a negative exponential function *EXP*(−*RAMP*({*slope*}, {*start*}, {*finish*})) was employed to superimpose the random variables *RANDOM UNIFIRM*({min}, {max}, {*seed*}) and describe the changes in the transportation and information delays. Three different delay levels were set to reflect the different emergency types and severities, as shown in Table 2.

**Table 2:** Transportation delay and information delay parameter settings.

**Delay level**	**Transportation delay**	**Information delay**	**Interpretation**
High	Exp [–RAMP(1/10,0,60)] * RANDOM NORMAL (0, 8, 4, 4, 0)	EXP [–RAMP(1/5,0,60)] * RANDOM NORMAL (0, 6, 3, 3, 0)	Traffic and communication are seriously affected or destroyed by the disaster
Medium	Exp (–RAMP(1/10,0,60)] * RANDOM NORMAL (0, 4, 2, 2, 0)	EXP [–RAMP(1/5,0,60)] * RANDOM NORMAL (0, 3, 1.5, 1.5, 0)	Traffic and communication are affected by the disaster and need some time to repair
Low	Exp [–RAMP(1/10,0,60)] * RANDOM NORMAL (0, 2, 1, 1, 0)	EXP (–RAMP(1/5,0,60)] * RANDOM NORMAL(0, 1.5, 0.75, 0.75, 0)	Roads and communications were slightly affected by the disaster

To study the impact of different government attitudes on NGO relief goods supply, the government's attitudes toward the NGOs' emergency relief were divided into three types: restriction, noninterference, and support; with the respective government support variable values set at −0.5, 0, and 0.5.

## 4. Results

### 4.1. Demand simulation

**Figure 10:**
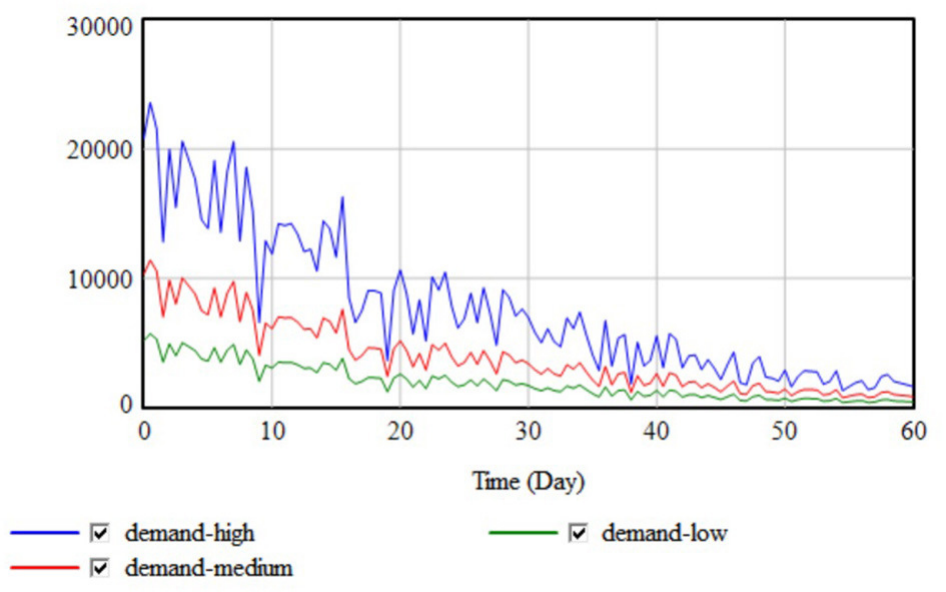
Demand simulation results.

Because different emergencies have different casualty and goods requirements, the needs were divided into low, medium, and high scenarios, the simulation results for which are shown in Figure 10.

The results showed that the three demand curves all peaked at 0 days and then gradually declined, which aligned with the post-disaster relief goods demand characteristics, that is, the more serious the disaster damage, the higher the demand, and the greater the demand fluctuations caused by secondary disasters.

In the COVID-19 case in Hubei, the wide variety of material types and specifications aggravated the information delay and often resulted in a mismatch between the supply and demand of relief goods. To simplify the model and focus on the supply chain characteristics, only a single type of relief goods was focused on.

### 4.2. Delay simulation

**Figure 11:**
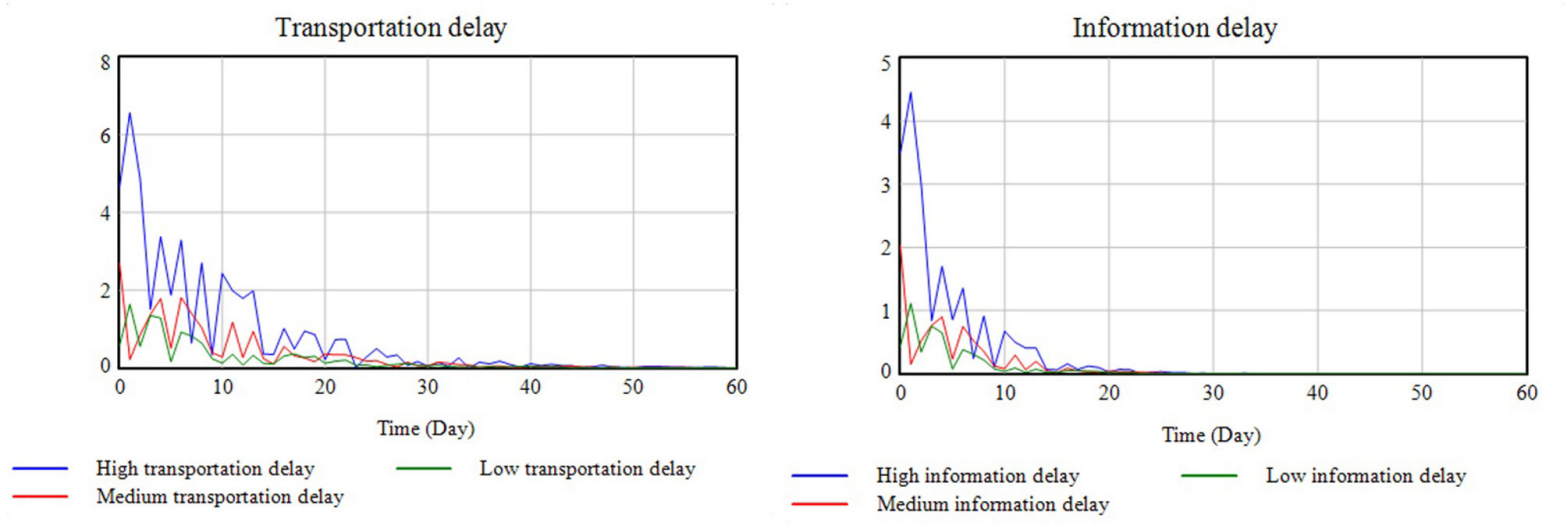
Delay simulation results.

Based on the data in Table 2, three different transportation and information delays were simulated, with the results shown in Figure 11.

It was found that the change trends in transportation and information delays were basically the same and were only affected by secondary disasters and post-disaster maintenance. However, because communication equipment is easy to replace and less difficult to maintain, the information delay decreased rapidly in the short term after the disaster while the transportation delay decreased more slowly. In the simulated case, COVID-19 was surmised to result in traffic restrictions and increased transportation delays.

The adequacy of NGO funds is also an important factor affecting delays. For example, NGOs can increase transportation capacity and reduce transportation delays by purchasing additional vehicles and can reduce information delays by increasing manpower or equipment inputs. However, during the investigation of the NGOs participating in supplying relief goods during the Hubei 2020 COVID-19 crisis, it was found that the most significant NGO problem was a lack of funds, which meant that the delays did not fall quickly to acceptable levels.

### 4.3. Supply simulation

(1) Impact of demand fluctuations in the pandemic areas

**Figure 12:**
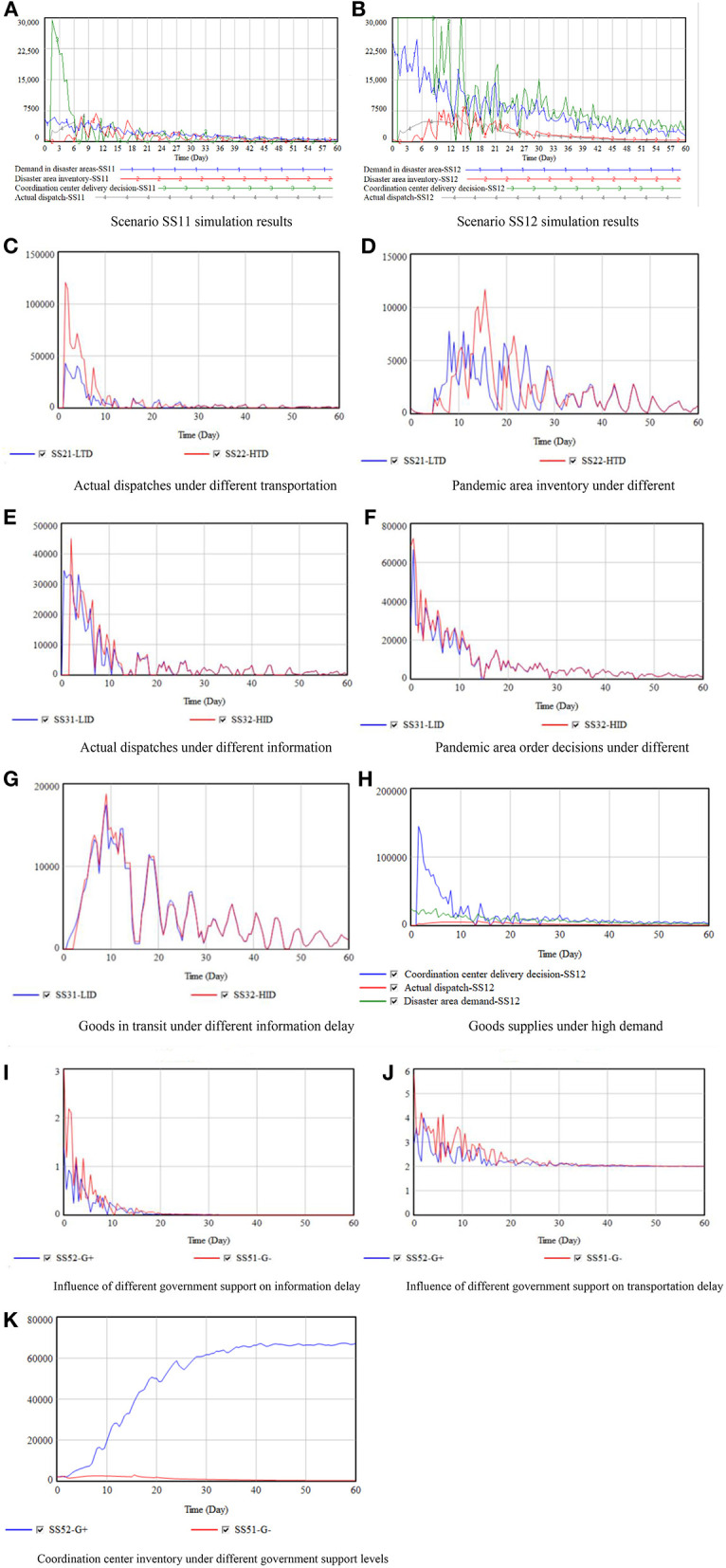
Goods supply simulation results.

The NGO relief goods supply simulation results under different dynamic demand levels in the pandemic area are shown in Figures 12A, B.

Due to the information (medium) and transportation (medium) delays, it was found that the NGOs received information about the relief goods demand in the pandemic area some time after the disaster, which meant the NGO materials were delayed in reaching the pandemic areas, as can be seen by the fluctuations in the curve. Further, because of the long-term nature of the demand and delays, the downstream supply chain fluctuations were amplified compared to the upstream fluctuations, that is, there was a bullwhip effect (55).

When white noise was added to the periodically fluctuating demand, the whole system fluctuated, as shown in Figures 12A, B. This was primarily because of the transportation delays as the demand lag resulted in a decline in inventory, which meant that the actual dispatch volume and demand satisfaction were no longer stable. Compared to the case without white noise (SS01), the total inventory means and variance, respectively increased by 5 and 150%.

With the dynamic changes in demand, the changes in the relief goods supply chain became more complex. When demand dynamically changes, the inventory fluctuates, and the greater the demand fluctuation level, the more intense the shock to the whole system (SS12), and the worse the material supply effect on the system. As shown in Table 3, the supply rate decreased from 57 to 28%, and the total inventory increased by 92%. Even though the demand fluctuated and decreased, the inventory first increased and then decreased, which was due to the following.

(i) The dynamic transportation and information delay changes meant that the expected inventory was no longer stable as the order information was no longer changing regularly with the demand.
(ii) The coordination center was short of goods. Immediately after the emergency, the materials demand reached its peak; however, the insufficient donations led to insufficient inventory at the coordination center to meet the order demands, that is, the system had short supplies in the early stages. As the attention toward the emergency increased, donations increased but demand decreased, which meant that there was a rise in the inventory.

(2) Delay impacts

**Table 3:** Supply simulation results.

**Scenario code**	**Exogenous variable**				**Evaluation indicator**			
	**Transportation delay (TD)**	**Information delay (ID)**	**Demand (D)**	**Government support (G)**	**Demand satisfaction rate**		**Total inventory**	
					**Mean value**	**Standard deviation**	**Mean value**	**Standard deviation**
SS01	TD = 2	ID = 0.5	Low	Non-interference (G = 0)	0.980	0.090	2.550	0.500
SS11	Medium	Medium	Low	Non-interference (G = 0)	0.572	0.337	0.364	0.341
SS12	Medium	Medium	High	Non-interference (G = 0)	0.278	0.183	0.687	0.606
SS21	Low	Medium	Medium	Non-interference (G = 0)	0.532	0.344	0.584	0.456
SS22	High	Medium	Medium	Non-interference (G = 0)	0.538	0.357	0.742	0.705
SS31	Medium)	Low	Medium	Non-interference (G = 0)	0.534	0.339	0.620	0.513
SS32	Medium	High	Medium	Non-interference (G = 0)	0.541	0.339	0.630	0.544
SS41	Low	Low	Medium	Non-interference (G = 0)	0.528	0.342	0.575	0.434
SS42	High	High	Medium	Non-interference (G = 0)	0.539	0.360	0.754	0.733
SS51	Medium	Medium	Medium	Restriction (G = −0.5)	0.286	0.161	0.362	0.320
SS52	Medium	Medium	Medium	Support (G = 0.5)	0.543	0.322	0.622	0.510

The impacts of the different delays on the relief goods supply system are shown in Figures 12C–G. Note that all other factors are assumed to be at a medium level,

Figure 12C shows that with an improvement in the transportation delay situation, the dispatch increased significantly in the early stage and was relatively consistent in the later stage. This was because the transportation delays decreased over time, which meant that the differences between the two scenarios were also gradually disappearing.

Figure 12D shows the impact of the different transportation delay levels on the inventory in the pandemic areas, indicating that the higher the delay level, the greater the inventory fluctuations, which resulted in goods shortages in the early stage. Table 3 shows that under severe transportation delays, the goods supply rate increased by 1%, and the total inventory increased by 27%.

The simulation results in Figure 12E show that as the information delay increased, the dispatch tended to have more irregular fluctuations, which was because the information delay affected the expected inventory in the pandemic areas, which then affected the order decisions. And the information delays affected the coordination center decisions by affecting the inventory and demand feedback from the pandemic areas.

The information delays also affected the coordination center information regarding the goods in transit. Figure 12G reflects the transit goods backlogs during transportation, which formed a negative feedback loop with the dispatch of the materials, prevented the coordination center from sending excessive materials, and avoided unnecessary congestion. When the information delays increased, the goods in transit also increased, which the results in scenarios SS31 and SS32 in Table 3 also confirmed.

(3) Insufficient supply impacts

In the early emergency relief stage, there was a very large demand for goods in the disaster area, but because inventory had been damaged, there were insufficient supplies. Consequently, it took time for the NGOs to collect the necessary goods, which when complete, were subject to information and transportation delays at the coordination center. In the 2020 Hubei COVID-19 crisis, because there were no reliable procurement channels and the procurement price and quality were difficult to control, it was difficult to convert the raised cash into relief goods. Therefore, the actual dispatch at the initial stage was far less than the demand and available transportation; however, there was better consistency in the latter stages, as shown in Figure 12H.

After February 8, 2020, the goods demand in the pandemic area were basically guaranteed, which was consistent with the simulation results. The 11 emergency scenarios established in this paper indicated that there would be no supply shortages after 20 days. Therefore, the initial goods inventory factor was the main factor affecting demand satisfaction.

(4) Impact of government support

Government support affects the relief goods supply chain by affecting the delay change rate. The government supports NGO emergency relief by sharing information and giving them traffic priority. Therefore, increased government support results in reduced transportation delays and an increase in the efficiency of the goods supply and emergency relief. Increased government support also improves the speed and accuracy of the NGOs' access to information; therefore, an increase in government support results in a reduction in transportation and information delays, as shown in Figures 12I, J.

Second, the government's strict EPC regulations can increase the public's attention on the disaster, which can assist the NGOs to raise funds and acquire materials, that is, the goods supply is promoted. Scenarios SS51 and SS52 (Figure 12K) show the situation when there is full government support and the effect on the goods raised by the NGOs; therefore, government support can have a significant impact on the effectiveness of NGO relief goods collection.

## 5. Discussion

### 5.1. Complementary to the simulation results

The results of the research team's investigation in Hubei further confirmed the above simulation results and made supplements to the results.

(1) Effects of delay and use of wechat

Through investigation, delay, especially information delay, had a great negative impact on the efficiency of relief goods supply after the lockdown of Hubei. For example, NGOs have no channels to obtain sufficient relief goods, and the types and models of medical supplies needed in epidemic areas are not clear.

Additionally, in the 2020 Hubei COVID-19 crisis, the popular Chinese social media app “WeChat” played an important demand assessment and goods distribution role, primarily because the NGOs lacked a unified relief goods supply information sharing platform. If this problem were solved, the information delay would have reduced and the transportation efficiency would have significantly improved.

(2) Government influence

Through investigation, the research team found that the NGOs assisting in the Hubei COVID-19 crisis had had significant difficulties because there were no stable, high-quality procurement channels. When many hospitals in Wuhan were reported to be short of medical supplies, the government provided a large number of supplies to NGOs to help alleviate the shortage of supplies in Hubei. So government support will be an effective way to solving this problem. However, the effect of government support is not always significant. For example, the supply effect under government support in scenario SS52 was worse than the supply effect under government non-interference in scenario SS11.

### 5.2. Policy suggestions

Based on the simulation results, some policy suggestions are given to provide a reference for governments and NGOs when participating in emergency relief goods supply emergencies in the future. When the scale and type of emergency change, the SD model could also be employed to simulate the changed conditions.

(1) Increase the standby emergency relief goods inventories in disaster-prone areas and enhance the relief goods storage resistance capacities to reduce the possible damage to emergency preparedness materials from a disaster. This could effectively deal with the relief goods shortages in the early disaster stage.
(2) A bullwhip effect exists in the supply of NGO relief goods, which becomes more intense and irregular when there are transport and information delays. Therefore, reducing these transportation and information delays is needed to stabilize the system and improve supply efficiency through such means as simplifying approval procedures, establishing integrated information mechanisms, and establishing special roads for emergency supply transport.
(3) In small and medium-sized emergencies, NGOs can play important emergency relief goods supply roles. Governments should support NGO emergency relief operations through policy encouragement and media publicity. However, in large-scale emergencies, the NGOs' roles are smaller as the government undertakes the main emergency relief tasks.

## 6. Conclusion

Taking the 2020 Hubei COVID-19 crisis as an example, this study conducted SD simulation research on NGO emergency goods supply, from which the following conclusions were drawn.

(1)Secondary disasters cause demand fluctuations in disaster areas, which also have a bullwhip effect on the post-disaster emergency supply chain. Coupled with insufficient funding, the whole system can have large irregular fluctuations.
(2)Dynamic transportation and information delays cause greater system fluctuations and uncertainties. Transportation delays affect inventory by delaying the circulation of goods and influencing decision maker expectations, and information delays affect inventory by influencing disaster information feedback and decision maker expectations. The superposition of these two delays can further amplify system fluctuations and uncertainties.
(3)In all simulated emergency scenarios, it was found that the supply of the goods was sufficient 20 days after the disaster and that the initial inventory at the coordination center played a decisive role in the initial emergency goods supply. If there is a lower initial inventory, there could be chaos or even increased casualties in disaster areas because of the lack of relief supplies.
(4)Government support can significantly reduce transportation and information delays, which in turn can reduce relief goods inventory fluctuations in disaster areas and increase the demand satisfaction rate. However, government support for NGO goods supply was found to be limited. When there is significant disaster damage, the government support impact on supply efficiency was found to be slight.

However, there were some limitations to this study. First, because of its “big government, small society” context, the state-civil society in China is special (46), which also means that NGOs are usually constrained, even though they were fully authorized during the COVID-19 crisis, exploratory research in combination with other countries is needed to assess. Second, the problem structures and environment may change on the different system levels, which means that different methodological choices could be made. However, this study did not conduct a detailed analysis of each system level, which could be a valuable future research direction. Third, long city lockdowns are a rare occurrence; therefore, this research needs to be extended to other types of emergency scenarios, such as local wars or other humanitarian crises, to examine the differences.

## Data availability statement

The raw data supporting the conclusions of this article will be made available by the authors, without undue reservation.

## Author contributions

YL determined the research contents and methods, reviewed the manuscript, and provided fund support for the research. YW prepared Figures 1–12. All authors wrote the main manuscript text.
